# Health literacy disparities in Thai university students: exploring differences between health science and non-health science disciplines

**DOI:** 10.1186/s12889-025-21761-0

**Published:** 2025-02-11

**Authors:** Thanyaporn Manochaiwuthikul, Amornrat Chaichutchouwakul, Nantakarn Yunan, Niwarin Winothai, Peewara Kanta, Ratana Sapbamrer

**Affiliations:** Department of Community Medicine, Faculty of Medicine, Chiang Mai University, 110 Inthavaroros Road, Sri Phum Subdistrict, Muang District, Chiang Mai, 50200 Thailand

**Keywords:** University, Student, Health literacy, Lifestyle, Behavior, Academic, Young adult

## Abstract

**Background:**

Health literacy (HL) in Thailand remains a significant issue, with a large portion of the population demonstrating limited HL, and limited research exists on specific determinants among Thai university students. Some culture shapes health decision-making and HL disparities within this group, highlighting the need to evaluate HL comprehensively. This study aims to assess and compare HL levels among health science and non-health science students, and identify key predictors associated with HL.

**Methods:**

A cross-sectional study was conducted among 1,647 Thai university students, comprising 676 health science and 971 non-health science students, recruited through multi-stage stratified random sampling. Data were collected using validated questionnaires that measured sociodemographic characteristics, academic background, lifestyle behaviors, and HL levels across three sub-domains, including healthcare, disease prevention, and health promotion. Statistical analyses included univariable and multivariable regression models, with significance set at *p*-value < 0.05.

**Results:**

Health sciences students demonstrated significantly higher HL scores across all sub-domains compared to non-health sciences students. Limited HL was found in 12.6% for health science students, and 28.4% for non-health science students. Key predictors of overall HL included gender, income, faculty of study, and lifestyle-related health behaviors. Female students showed higher total HL (β = 1.41; 95% CI: 0.62, 2.21), as did those with higher income (β = 1.28; 95% CI: 0.76, 1.80). Students enrolled in health sciences programs had higher HL scores (β = 2.86; 95% CI: 2.07, 3.64). Lifestyle behaviors also correlated with HL, with non-smoking (β = -0.58; 95% CI: -1.03, -0.13), no alcohol consumption (β = -0.45, 95%CI: -0.89, -0.01), and regular physical activity (β = 0.35; 95% CI: 0.04, 0.66). Consumption of sweet foods/drinks was inversely related to HL scores (β = -0.87; 95% CI: -1.49, -0.26).

**Conclusions:**

HL among Thai university students varies significantly by sociodemographic factors, academic background, and lifestyle behaviors. Health sciences students exhibited stronger HL skills, underscoring the importance of integrating health education into non-health sciences curricula. Targeted HL interventions are recommended for non-health sciences students, males, and individuals from lower-income backgrounds to enhance health decision-making and reduce HL disparities. These findings have implications for policy and practice, emphasizing the integration of HL modules into university curricula, health promotion campaigns, and the provision of accessible health services to foster an inclusive and health-literate student population.

**Supplementary Information:**

The online version contains supplementary material available at 10.1186/s12889-025-21761-0.

## Introduction

Health literacy (HL), defined as the ability to access, comprehend, appraise, and apply health-related information, is a critical competency that enables individuals to make informed health decisions. Increasingly, HL is recognized as a determinant of health outcomes, as higher levels contribute to more effective health risk management, adherence to preventive measures, and greater engagement with healthcare services [1, 2]. A comparative analysis of HL between Asian and European populations reveals that HL scores tend to be lower among Asian individuals compared to their European counterparts. Findings from the European HL Survey (HLS-EU) indicate HL scores ranging from 29.6 to 34.4 in Asian populations, while European populations show scores between 30.5 and 37.06 [3, 4]. Studies conducted in Asia have identified significant associations between HL levels and factors such as age, education, health status, income, and residential area [3]. In contrast, a European study found that age, gender, educational attainment, financial deprivation, and social status were significant predictors of HL levels [4]. Low HL has substantial economic and social consequences, contributing to increased healthcare costs, poorer health outcomes, widening health disparities, and a greater burden on public health systems [5–7].

For young adults, particularly university students, HL is essential as they transition to independent living, encountering new health challenges and heightened personal responsibility for health maintenance [8]. Additionally, university students represent a unique demographic that faces lifestyle changes, academic pressures, and diverse health information sources, all of which influence their HL [9, 10]. Prior studies suggest that students’ HL is shaped by a range of factors, including sociodemographic characteristics, academic background, cultural context, and lifestyle behaviors such as smoking, alcohol consumption, dietary habits, and physical activity. However, research in this area has yielded mixed findings, with some studies identifying strong associations between HL and these factors, while others report limited or conflicting outcomes [11].

In Thailand, HL across all age groups is a critical issue. Previous research reveals that a significant portion of the Thai population has only a fair level of HL (58.9%), with a notable percentage demonstrating low HL (16.8%) [12]. While several studies in Thailand have focused on developing and examining HL among specific risk groups; however, there is a notable lack of research addressing general HL within the general population [12–14]. Despite the recognized importance of HL in shaping long-term health outcomes, there remains a substantial knowledge gap regarding the specific determinants of HL among Thai university students. Additionally, there are limited exploration of HL disparities between academic disciplines, such as health science and non-health science students. This gap is notable given the differences in exposure to health-related content and learning environments between these groups. The lack of comparative studies in this area leaves a critical gap in identifying tailored interventions to address HL disparities. Addressing this research gap is essential to better understand how academic backgrounds influence HL and to design effective strategies to bridge these disparities.

Socio-demographic and behavioral determinants play a pivotal role in shaping HL in the Thai context. Factors such as gender, income, parental education, and lifestyle behaviors directly influence how individuals access and utilize health information. These factors are especially relevant in Thailand, where socioeconomic inequalities persist and contribute to health disparities. To address HL needs in young adult populations, the Department of Health, Ministry of Thailand, introduced the “Health Media for School-Aged Children and Adolescents” initiative, which provides accessible, age-appropriate media for children and adolescents in primary and secondary schools. However, this initiative does not extend to young adults [15]. Additionally, cultural factors in Thailand strongly impact young adults’ HL. Traditional beliefs often conflict with modern medical advice. Taboo topics like mental and sexual health discourage help-seeking, while reliance on peers and unverified sources risks misinformation. Complex medical language also hinders understanding, underscoring the need for culturally sensitive interventions to improve HL [16–18]. By understanding these cultural nuances, targeted interventions can be designed to address the unique barriers faced by young adults in Thailand, promoting equitable health outcomes.

This study aimed to bridge this gap by comparing HL levels between health science and non-health science students. The study also aims to identify key factors influencing HL, such as sociodemographic characteristics, academic background, and lifestyle related health behaviors. Ultimately, the findings from this study are expected to offer valuable insights for policy and practice in university health promotion. A deeper understanding of the factors influencing HL among Thai university students will support the development of health interventions tailored to the specific needs of this demographic, enabling them to make informed health choices and contributing to improved public health outcomes in university settings.

## Methods

### Setting, study design, and participants

Between July and September 2024, a cross-sectional study was conducted to assess and compare HL levels among health science and non-health science students, and to identify key predictors across three sub-domains, including healthcare, disease prevention, and health promotion. The study was carried out at a government university in northern Thailand during the 2024 academic year. This study was conducted at the largest university in northern Thailand, which has the highest student enrolment in the region, encompassing 21 faculties and a diverse student body. This ensures the university’s representativeness of the broader student population in the northern region. The choice of a single institution was made to leverage its comprehensive academic and demographic diversity while ensuring feasibility for data collection. Therefore, this approach can generalize the findings to students in northern regions or universities. The university comprises 21 faculties, with six in health sciences and 15 in non-health sciences. The total undergraduate enrollment was 27,734 students, with 5,056 in health science faculties and 22,678 in non-health science faculties. The inclusion criteria for the study were as follows: (1) undergraduate students actively enrolled in their first to fourth year of study; (2) registered in a regular academic program during the 2024 academic year; and (3) proficient in reading and writing in Thai. Students who did not complete all questions in the questionnaire were excluded. A multi-stage stratified random sampling approach was employed. Initially, the student population was divided into two strata: health science and non-health science faculties. Subsequently, 10 faculties were randomly selected, with three from health sciences (Medicine, Nursing, and Medical Technology) and seven from non-health sciences (Engineering, Science, Architecture, Law, Humanities, Fine Arts, and Political Science). This process ensured representation across disciplines. The research team distributed invitation posters and online questionnaires through each faculty’s student union to publicize and invite student participation in the study.

Utilizing the EpiInfo program, the required sample size was calculated based on an expected frequency of 50% for HL, a margin of error of 2.5%, and a planned statistical power of 80%. This calculation yielded a minimum sample size of 1,455 participants. Of the 2,000 students initially invited, 1,980 participated in the survey by answering the questionnaire, and 1,647 provided complete responses, yielding a response rate of 82.4%. A total of 333 participants (16.8% of those who answered the questionnaire) were excluded due to incomplete responses. The final sample included 676 students from health science faculties and 971 from non-health science faculties, enabling a comprehensive assessment of HL levels and their determinants across diverse undergraduate groups within the university.

### Questionnaire

Participants were invited to complete an online structured questionnaire, which took approximately 10 min to finish. The questionnaire comprised five sections: sociodemographic information, academic background details, lifestyle-related health behaviors, healthcare service utilization, and health literacy assessment. Sociodemographic data included age (years), gender (male or female), religion (Buddhist or others), relationship status (single or have boy/girlfriend), monthly income (< 150 USD/month, 150–300 USD/month, or > 300 USD/month), parental education (primary school or lesser, secondary school, Bachelor degree or higher), living status (with roommate, alone, or with family), hometown status (rural or urban), co-morbidity (yes or no), and history illness in the past six months (yes or no). Academic data included year of study (1^st^, 2^nd^, 3^rd^, or 4^th^ year), cumulative GPAX (< 2.50, 2.51–3.50, or > 3.51). Lifestyle-related health behavior data included smoking (never, 1–2 days/week, 3 days/week, 4–5 days/week, or 6–7 days/week), alcohol consumption (never, 1–2 days/week, 3 days/week, 4–5 days/week, or 6–7 days/week), physical activity (never, 1–2 days/week, 3 days/week, 4–5 days/week, or 6–7 days/week), and dietary habits of fatty foods, sweet foods/drinks, or salty foods (never, 1–3 days/week, or 4–7 days/week). Utilization of Healthcare service information included actions taken when illness (purchase medication, visit physician, or leave it untreated), hospital use (university hospital, government hospital, clinic, or private hospital), and reason to choose hospital (acceptance insurance, convenient location, quick service, good service). This comprehensive questionnaire was designed and developed to capture a wide range of factors potentially influencing health literacy among the student population (Supplementary 1).

The European Health Literacy Survey Questionnaire (HLS-EU-Q47) was used to measure HL. It consisted of 47 items that evaluate an individual’s ability to access, understand, appraise, and apply health-related information in three sub-domains, including healthcare, disease prevention, and health promotion. each item was rated on a 4-point Likert scale, such as very difficult, difficult; easy, and very easy [19]. To standardize HL scores on a scale from 0 to 50, the following formula is applied:

$$\mathrm{HL}\;\mathrm{index}\;=\;(\mathrm{mean}\;-1)\;\mathrm x\;(50/3)$$

In this formula, the “Mean Score” represents the average response in each index.

Based on the HL Index, health literacy was categorized into four levels, including inadequate HL (0–25 scores), problematic HL (> 25–33 scores), sufficient HL (> 33–42 scores), and excellent HL (> 42–50 scores). For identifying vulnerable or target groups, the “inadequate” and “problematic” HL categories were combined into a single group termed “limited HL,” encompassing scores from 0 to 33. This classification system facilitates the identification of individuals or populations that may benefit from targeted health literacy interventions.

The HLS-EU-Q47 questionnaire underwent a forward-only translation from English to Thai by two native Thai researchers fluent in English (RS and TM). Three additional researchers (NY, NW, and AC) reviewed and edited the Thai version. Validity was further assessed by five researchers (RS, TM, NY, NW, AC), and pilot testing was conducted with five participants. After addressing minor wording, grammatical and typographical errors, TM prepared the final version. The questionnaire’s validity and reliability were evaluated before use, with all items achieving an Index of Congruence (IOC) score above 0.5. Cronbach’s alpha coefficients were 0.85 for the rating scale and 0.86 for the Thai version of the HLS-EU-Q47, indicating high internal consistency.

### Ethical approval

This study was approved by the research Ethics Committee of Faculty of Medicine, Chiang Mai University, Thailand (No.137/2024, Approval date 28 March 2024). Participants' anonymity and confidentiality were ensured throughout the study. Identifiable information was not collected, and responses were anonymized during data collection and analysis. Data were stored securely on a password-protected server accessible only to authorized research team members. Informed consent was obtained from all participants before they accessed the online questionnaire. A detailed information sheet was provided at the beginning of the survey, explaining the study objectives, procedures, potential risks, and benefits. Participants were required to indicate their consent electronically before proceeding with the questionnaire. The online questionnaire was administered using the REDCap platform, which provided a secure and user-friendly interface for data collection. The platform settings were configured to prevent the collection of IP addresses, ensuring participant anonymity.

### Statistical analysis

Descriptive statistics, including mean and standard deviation (SD.) for continuous variables and frequency (n) and percentage (%) for categorical variables, were calculated to summarize sociodemographic characteristics, academic information, and lifestyle-related health behaviors of the study participants. Differences between health science and non-health science students across these variables were evaluated using independent t-tests for continuous variables and chi-square tests for categorical variables.

HL levels, total and within sub-domains (healthcare, disease prevention, and health promotion), were assessed as continuous outcomes, with comparisons between health science vs. non-health science students conducted through independent t-tests. To categorize HL, the European Health Literacy Survey Questionnaire (HLS-EU-Q47) was scored, with sub-domain scores analyzed individually.

Univariable and multivariable linear regression models were applied to examine factors associated with HL scores. Univariable analyses were conducted to identify factors with potential associations with HL outcomes. Variables that were statistically significant in the univariable analyses (*p* value < 0.05) were subsequently included in the multivariable linear regression models. This step ensured that only statistically significant predictors were retained for further analysis. These variables comprised sociodemographic characteristics (gender, age, income, parental education), academic parameters (faculty and GPAX), and health-related behaviors (smoking, alcohol consumption, and dietary habits). Subsequently, multivariable models were constructed to assess these relationships while adjusting for potential confounders. The variance inflation factor (VIF) threshold of < 10 was applied to confirm the absence of multicollinearity, ensuring that predictor variables were not highly correlated. The final model retained variables significant at a *p*-value of < 0.05.

## Results

### Sociodemographic, academic background, and lifestyle behaviors

A total of 333 participants (16.8% of the initial sample) were excluded due to incomplete responses. Compared to included participants, excluded participants were more likely to be male (47.6% vs. 33.7%), from non-health science faculties (76.7% vs. 58.9%), and had lower reported GPAX scores (< 2.50 in 34.2% of exclusions vs. 20.0% of inclusions). These differences highlight potential systematic disparities between the excluded and included groups.

The sociodemographic characteristics of health sciences and non-health sciences students (*n* = 1,647) displayed several significant differences. The average age of health sciences students was slightly higher (20.2 ± 1.7 years) compared to non-health sciences students (19.9 ± 1.3 years), with a statistically difference. Gender distribution revealed that a higher percentage of health sciences students were female (78.0%) compared to non-health sciences students (58.2%). Health sciences students had a higher prevalence of parents with a bachelor’s degree or higher (60.9%) compared to non-health sciences students (50.5%). The relationship status, religion, monthly income levels, living and hometown status showed no statistically significant differences between groups, indicating similar distributions.

**Fig. 1:**
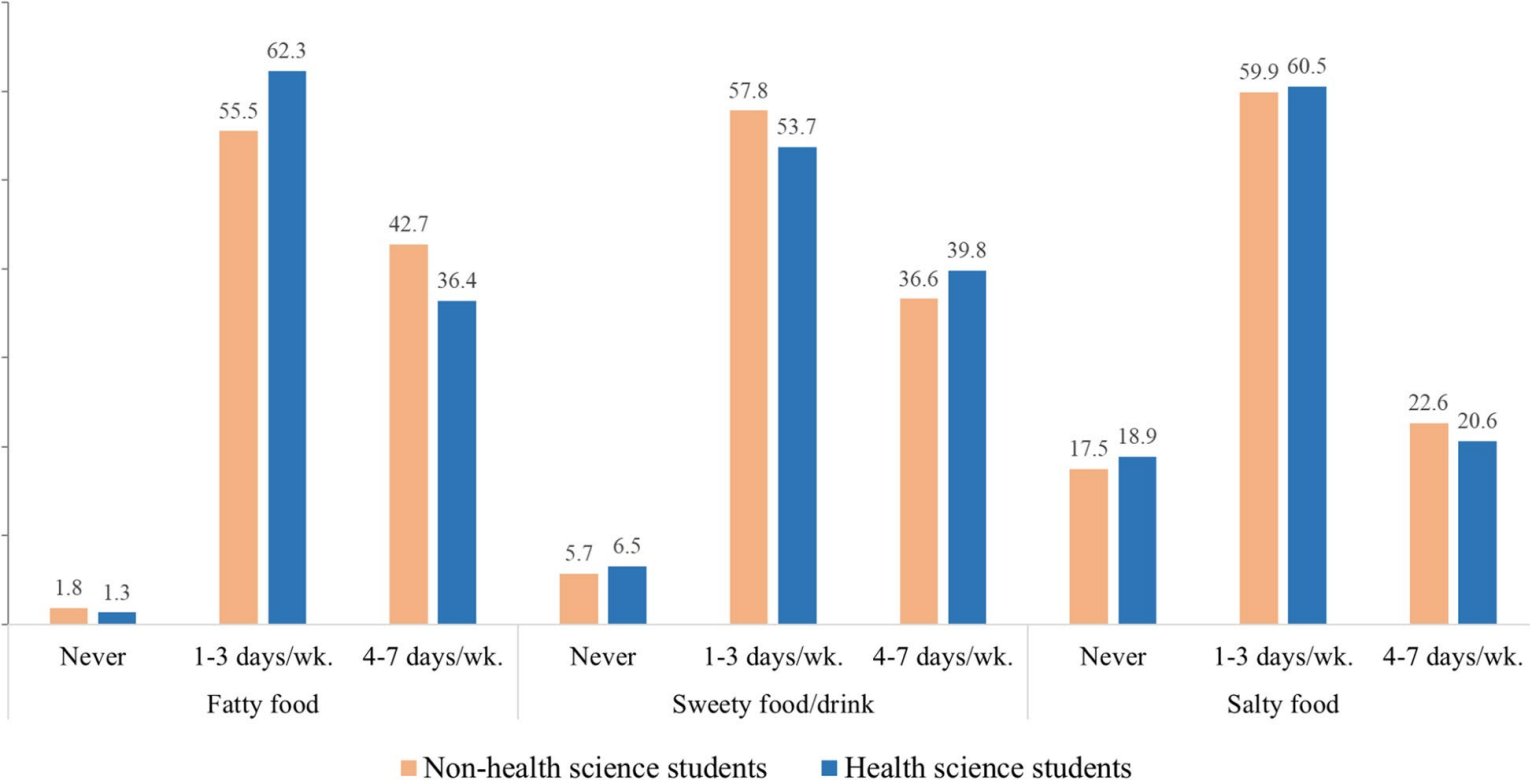
Dietary habits between health science and non-health science students

Regarding lifestyle-related health behaviors, smoking and alcohol consumption patterns differed notably. Health sciences students reported a significantly lower prevalence of smoking across all frequency categories, with 95.7% never having smoked compared to 86.4% of non-health sciences students. Additionally, a significantly higher percentage of health sciences students abstained from alcohol consumption (62.1% vs. 38.4%). Physical activity frequency, comorbidity, and history illness showed no significant differences between groups, suggesting similar patterns in these health behaviors (Table 1). In terms of dietary habits, non-health science students reported more frequent consumption (4–7 days per week) of fatty and salty foods, whereas health science students reported a higher frequency of sweet foods/drinks consumption. However, the overall differences in consumption between health science and non-health science students were not statistically significant (Fig. 1).

In terms of healthcare utilization, health sciences students reported a significantly a greater tendency to visit a physician when illness (75.1% for health science students and 69.9% for non-health science students), and preference for government hospitals was higher among health sciences students (65.8% for health science students and 60.8% for non-health science students). Reasons for hospital choice highlighted significant differences; health sciences students were more likely to choose facilities based on insurance acceptance and quick service (Table 1).

**Table 1:** Sociodemographic and other information among health sciences and non-health science students (*n* = 1,647)

Parameters		Total (*n* = 1647)	Heath science students (*n* = 676)	Non-health science students (*n* = 971)	*p* value
Age		20 *±* 1.5	20.2 *±* 1.7	19.9 *±* 1.3	< 0.001**
Gender	Male	555 (33.7)	149 (22.0)	406 (41.8)	< 0.001**
	Female	1092 (66.3)	527 (78.0)	565 (58.2)	
Religion	Buddhist	1547 (93.9)	633 (93.6)	914 (94.1)	0.682
	Others	100 (6.1)	43 (6.4)	57 (5.9)	
Relationship status	Single	1149 (69.8)	475 (70.3)	674 (69.4)	0.711
	Boyfriend/girlfriend	498 (30.2)	201 (29.7)	297 (30.6)	
Monthly income	< 150 USD/month	592 (35.9)	240 (35.5)	352 (36.3)	0.832
	150–300 USD/month	819 (497)	335 (49.6)	484 (49.8)	
	> 300 USD/month	236 (14.3)	101 (14.9)	135 (13.9)	
Parental education	Primary school or lesser	246 (14.9)	84 (12.4)	162 (16.7)	< 0.001**
	Secondary school	499 (30.3)	180 (26.6)	319 (32.9)	
	Bachelor degree or higher	902 (54.8)	412 (60.9)	490 (50.5)	
Living status	With roommate	755 (45.8)	310 (45.9)	445 (45.8)	0.750
	Alone	703 (42.7)	293 (43.3)	410 (42.2)	
	With family	189 (11.5)	73 (10.8)	116 (11.9)	
Hometown status	Rural community	1012 (61.4)	399 (59.0)	613 (63.1)	0.092
	Urban community	635 (38.6)	277 (41.0)	358 (36.9)	
Comorbidity		159 (9.7)	71 (10.5)	88 (9.1)	0.330
History illness in past 6 months		920 (55.9)	362 (53.6)	558 (57.5)	0.115
Smoking	Never	1486 (90.2)	647 (95.7)	839 (86.4)	< 0.001**
	1-2days/week	42 (2.6)	8 (1.2)	34 (3.5)	
	3days/week	40 (2.4)	8(1.2)	32 (3.3)	
	4-5days/week	24 (1.5)	3 (0.4)	21 (2.2)	
	6-7days/week	55 (3.3)	10 (1.5)	45 (4.6)	
Alcohol consumption	Never	793 (48.1)	420 (62.1)	373 (38.4)	< 0.001**
	1-2days/week	611 (37.1)	218 (32.2)	393 (40.5)	
	3days/week	158 (9.6)	29 (4.3)	129 (13.3)	
	4-5days/week	51 (3.1)	4 (0.6)	47 (4.8)	
	6-7days/week	34 (2.1)	5 (0.7)	29 (3.0)	
Physical activity	Never	395 (24.0)	171 (25.3)	224 (23.1)	
	1-2days/week	590 (35.8)	245 (26.2)	345 (35.5)	0.174
	3days/week	339 (20.6)	141 (20.9)	198 (20.4)	
	4-5days/week	205 (12.4)	83 (12.3)	122 (12.6)	
	6-7days/week	118 (7.2)	36 (5.3)	82 (8.4)	
Year of study	1st year	428 (26.0)	164 (24.3)	264 (27.2)	0.020*
	2nd year	426 (25.9)	157 (23.2)	269 (27.7)	
	3rd year	372 (22.6)	160 (23.7)	212 (21.8)	
	4th year	421 (25.6)	195 (28.8)	226 (23.3)	
GPAX	< 2.50	329 (20.0)	40 (5.9)	289 (29.8)	< 0.001**
	2.51–3.50	736 (44.7)	305 (45.1)	431 (44.4)	
	> 3.51	582 (35.3)	331 (49.0)	251 (25.8)	
How usually do when you sick	Purchase medication	1338 (81.2)	557 (82.4)	781 (80.4)	0.315
	Visit a physician	1187 (72.1)	679 (75.1)	679 (69.9)	0.020*
	Leave it untreated	804 (48.8)	343 (50.7)	500 (51.5)	0.763
Hospital	University hospital	1166 (70.8)	476 (70.4)	690 (71.1)	0.777
	Government hospital	1035 (62.8)	445 (65.8)	590 (60.8)	0.036*
	Clinic	761 (46.2)	145 (21.4)	214 (22.0)	0.776
	Private hospital	359 (21.8)	286 (42.3)	475 (48.9)	0.008**
Reason to choose hospital for treatment	Acceptance insurance	1243 (75.5)	532 (78.7)	711 (73.2)	0.011*
	Convenient location	1060 (64.4)	444 (65.7)	616 (63.4)	0.350
	Quick service	984 (59.7)	382 (56.5)	602 (62.0)	0.025*
	Good service	652 (39.6)	262 (38.8)	390 (40.2)	0.566

*p value < 0.05

** *p* value < 0.01

### HL levels in university students

**Fig. 2:**
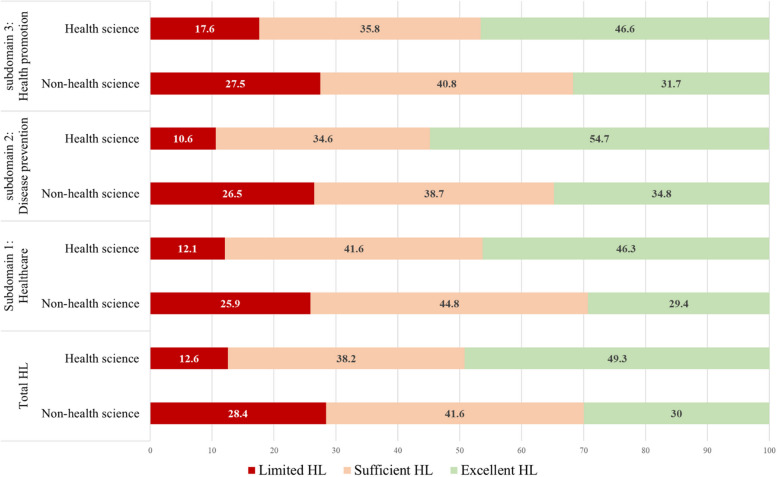
Health literacy categories and subdomains among health sciences and non-health science students

The analysis of HL among health sciences and non-health sciences students (*n*=1,647) across three sub-domains revealed significant differences in scores between these two groups. In the healthcare sub-domain, health sciences students scored significantly higher (40.6 ± 6.83) than non-health sciences students (37.18 ± 7.81). In the disease prevention sub-domain, health sciences students also demonstrated significantly higher scores (41.92 ± 7.47) compared to non-health sciences students (37.59 ± 8.70). For the health promotion sub-domain, health sciences students also scored significantly higher (40.39 ± 7.97) than non-health sciences students (37.18 ± 8.79) (Table 2). Considering with individual HL items, the three lowest-scoring questions are: question 47, “Participate in activities that improve health and well-being in your university?” (Mean ± SD = 2.95 ± 0.97); question 34, “Find information on how your friends could adopt healthier behaviors?” (Mean ± SD = 2.99 ± 0.92); and question 12, “Evaluate the reliability of illness-related information in the media?” (Mean ± SD = 3.03 ± 0.82) (Supplementary 2). When assessing HL using the three cut-off points, non-health science students demonstrated a significantly higher percentage of limited HL across all sub-domains compared to health science students (Figure 2).

**Table 2:** Health literacy scores among health sciences and non-health science students

HL Domains		Total (*n* = 1,647)	Health science students (*n* = 676)	Non-health science students (*n* = 971)	*p* value
**Sub-domain1: Healthcare**		**38.6 ± 7.6**	**40.60 ± 6.83**	**37.18 ± 7.81**	< 0.001**
	Accessing	36.9 ± 9.0	38.9 ± 8.4	35.4 ± 9.2	< 0.001**
	Understanding	39.7 ± 9.0	41.7 ± 8.1	38.3 ± 9.3	< 0.001**
	Appraising	36.5 ± 9.9	38.6 ± 9.4	35.0 ± 10.0	< 0.001**
	Applying	41.3 ± 8.4	43.1 ± 7.4	40.0 ± 8.9	< 0.001**
**Sub-domain2: Disease prevention**		**39.4 ± 8.5**	**41.92 ± 7.47**	**37.59 ± 8.70**	< 0.001**
	Accessing	39.2 ± 9.6	41.6 ± 8.6	37.5 ± 9.9	< 0.001**
	Understanding	43.3 ± 8.8	45.6 ± 7.3	41.7 ± 9.4	< 0.001**
	Appraising	37.8 ± 10.5	40.8 ± 9.5	35.8 ± 10.7	< 0.001**
	Applying	38.2 ± 10.8	40.5 ± 9.6	36.5 ± 11.3	< 0.001**
**Sub-domain3: Health promotion**		**38.5 ± 8.6**	**40.39 ± 7.97**	**37.18 ± 8.79**	< 0.001**
	Accessing	37.7 *±* 10.1	39.5 *±* 9.6	36.4 *±* 10.3	< 0.001**
	Understanding	40.5 *±* 9.1	42.9 *±* 7.9	38.8 *±* 9.5	< 0.001**
	Appraising	40.9 *±* 9.8	42.9 *±* 9.0	39.5 *±* 10.0	< 0.001**
	Applying	35.8 *±* 11.5	37.1 *±* 11.4	34.9 *±* 11.5	< 0.001**

** *p* value < 0.01

### Factors associated with HL in university students

The univariable analysis identified several factors significantly associated with HL among university students across the total score and three sub-domains. The analysis of sociodemographic parameters indicated that female students had higher HL scores than male students across all sub-domains. Additionally, students with a higher monthly income (> 300 USD/month) scored higher in HL across all sub-domains compared to those with lower incomes. Furthermore, students whose parents held a bachelor’s degree or higher demonstrated greater HL than those from other educational backgrounds. In terms of lifestyle-related health parameters, students who reported never smoking or consuming alcohol exhibited higher HL scores across all sub-domains compared to those with frequent usage. Physical activity also showed a positive association with HL, as students who engaged in regular exercise scored significantly higher than those with lower levels of physical activity. Regarding dietary habits, students who rarely or never consumed fatty foods, sweets, or salty foods achieved higher HL scores across all sub-domains compared to those with frequent consumption of these dietary items. With respect to academic parameters, students with a cumulative GPAX above 3.51 reported significantly higher HL compared to those with lower GPAX. However, no statistically significant differences in HL were observed based on the year of study (Supplementary 3).

The multivariable analysis identified several significant factors associated with HL among university students across the total score and three sub-domains. For sociodemographic factors, gender and monthly income were positively associated with HL across all sub-domains. Female students and students in higher income brackets had higher total HL scores (β = 1.41, 95% CI = 0.62, 2.21 for gender, and β = 1.28, 95%CI = 0.76, 1.80 for monthly income). Age and parental education level showed no significant associations with HL scores. Regarding with lifestyle-related health behavior factors, total HL was negatively associated with smoking (β = -0.58, 95%CI = -1.03, -0.13), alcohol consumption (β = -0.45, 95%CI = -0.89, -0.01), and eating sweet foods/drinks (β = -0.87, 95%CI = -1.49, -0.26), but positively associated with physical activity (β = 0.35, 95%CI = 0.04, 0.66). Among academic factors, students enrolled in health sciences faculties demonstrated higher total HL scores compared to those in non-health sciences faculties (β = 2.86, 95%CI = 2.07, 3.64), with elevated scores observed across all sub-domains. However, cumulative GPAX did not exhibit a significant association with HL in this analysis (Table 3).

**Table 3:** Multivariable analysis for investigating factors associated with health literacy in university students

Factors	β *±* SE. (95%CI)			
	Total score of HL	HL in Sub-domain1: Healthcare	HL in Sub-domain2: Disease prevention	HL in Sub-domain3: Health promotion
**Sociodemographic factor**				
Age	−0.05 *±* 0.13 (−0.30, 0.20)	0.12 *±* 0.13 (−0.14, 0.38)	−0.03 *±* 0.15 (−0.32, 0.25)	−0.247 *±* 0.150 (−0.542, 0.049)
Gender	1.41 *±* 0.40 (0.62, 2.21)**	1.62 *±* 0.42 (0.80, 2.44)**	1.69 *±* 0.46 (0.78, 2.60)**	0.945 *±* 0.478 (0.008, 1.882)*
Monthly income	1.28 *±* 0.27 (0.76, 1.80)**	1.22 *±* 0.28 (0.67, 1.76)**	1.41 *±* 0.31 (0.81, 2.01)**	1.226 *±* 0.315 (0.609, 1.843)**
Parental education	0.20 *±* 0.25 (−0.28, 0.68)	0.08 *±* 0.25 (−0.42, 0.57)	0.44 *±* 0.28 (−0.11, 0.99)	0.100 *±* 0.290 (−0.469, 0.669)
**Academic factors**				
Faculty study	2.86 *±* 0.40 (2.07, 3.64)**	2.54 *±* 0.41 (1.72, 3.35)**	3.28 *±* 0.46 (2.38, 4.19)**	2.784 *±* 0.474 (1.855, 3.713)**
GPAX	0.30 *±* 0.27 (−0.23, 0.83)	0.28 *±* 0.28 (−0.27, 0.83)	0.55 *±* 0.31 (−0.06, 1.16)	0.085 *±* 0.319 (−0.541, 0.711)
**Lifestyle factor**				
Smoking	−0.58 *±* 0.23 (−1.03, −0.13)*	−0.73 *±* 0.24 (−1.19, −0.26)**	−0.48 *±* 0.26 (−0.99, 0.04)	−0.531 *±* 0.269 (−1.059, −0.004)*
Alcohol consumption	−0.45 *±* 0.22 (−0.89, −0.01)*	−0.40 *±* 0.23 (−0.85, 0.05)	−0.49 *±* 0.26 (−0.99, 0.01)	−0.458 *±* 0.263 (−0.975, 0.059)
Physical activity	0.35 *±* 0.16 (0.04, 0.66)*	0.10 *±* 0.16 (−0.22, 0.42)	0.07 *±* 0.18 (−0.29, 0.42)	0.865 *±* 0.186 (0.500, 1.229)*
Eating fatty foods	−0.41 *±* 0.35 (−1.10, 0.28)	−0.22 *±* 0.36 (−0.93, 0.50)	−0.79 *±* 0.40 (−1.59, 0.00)*	−0.239 *±* 0.417 (−1.056, 0.578)
Eating sweet foods/drinks	−0.87 *±* 0.31 (−1.49, −0.26)**	−0.77 *±* 0.33 (−1.41, −0.13)*	−0.68 *±* 0.36 (−1.38, 0.03)	−1.163 *±* 0.372 (−1.893, −0.433)**
Eating salty foods	−0.19 *±* 0.29 (−0.76, 0.38)	−0.23 *±* 0.30 (−0.82, 0.35)	−0.10 *±* 0.33 (−0.75, 0.55)	−0.235 *±* 0.341 (−0.904, 0.434)

* *p* value < 0.05

** *p* value < 0.01

## Discussion

Our study reveals that sociodemographic and economic factors, particularly gender, and financial status, influence HL levels in university students. These findings align with existing literature suggesting that gender differences in HL may be attributed to social and cultural factors that encourage women to be more proactive in managing health information and engaging with healthcare services [11, 20–23]. Lee et al. [22] found that higher HL in women was linked to higher education and consistent healthcare access, while in men, it was associated with being unmarried and better self-rated health. Women’s frequent healthcare interactions, often tied to caregiving roles, may enhance their HL. Additionally, higher depression and chronic illness rates may drive greater healthcare engagement. These gender-specific factors highlight the need for tailored HL programs to promote health across genders. Our findings reveal that female students and those from higher-income backgrounds exhibited significantly higher HL scores across all sub-domains. These findings align with existing literature suggesting that gender differences in HL may be attributed to social and cultural factors that encourage women to be more proactive in managing health information and engaging with healthcare services.

Socioeconomic factors also showed a significant association with HL, with students from higher-income backgrounds exhibiting greater HL. It is likely that individuals from higher-income groups have more opportunities to engage in health-promoting activities and access preventive care, further supporting their HL [24, 25]. In contrast, individuals with poor financial resources often face limited access to education, healthcare services, and health information. These constraints can hinder their ability to understand and utilize health-related information effectively. Economic constraints may also lead to prioritizing immediate financial needs over health behaviors, further limiting engagement in health-promoting activities [26]. Financial resources also impact access to health-enhancing environments, such as areas with more healthcare facilities, recreational spaces, and nutritional options [27–29]. Therefore, addressing these disparities may require targeted HL interventions, financial support for low-income students, and culturally tailored programs reflecting gender norms. Improving school environments could also improve HL by fostering students’ self-efficacy in managing their health [30], contributing to better health outcomes across diverse demographics.

Academic factors are also significant determinants of HL. In this study, students in health sciences exhibited notably higher HL across all sub-domains compared to their counterparts in non-health sciences, likely due to greater access to and understanding of health information, as well as a vested interest in health promotion [11, 31]. These findings highlight the need for targeted interventions to improve health-related knowledge across academic disciplines, with a particular focus on non-health science students. Nevertheless, our study revealed that both groups (health and non-health sciences students) struggle in specific areas, including active participation in health-promoting activities, seeking information to support healthier behaviors among peers, and critically assessing the reliability of illness-related information in media sources. These gaps may be attributed to limited student engagement in campus health initiatives, insufficient confidence in identifying credible sources, and challenges in evaluating health information across diverse media channels. To address these issues, integrating HL programs into academic curricula, promoting HL awareness and resources, and strengthening media literacy skills are essential steps [11, 31].

Lifestyle-related health behaviors play a critical role in HL outcomes. Our study identified a significant association between smoking and lower total HL scores, as well as healthcare and health promotion sub-domains. In contrast, alcohol consumption was linked to lower total HL scores but did not show a significant association with any specific HL sub-domains. It might be due to HL’s direct influence on adolescents’ abilities to understand and manage health risks associated with smoking, fostering caution around tobacco use. Conversely, alcohol consumption appears more strongly influenced by cultural and social factors, such as traditional norms, peer influence, and early exposure within social environments, that may supersede the influence of HL in moderating drinking behaviors. Adolescents with lower HL may struggle to comprehend and apply health information, increasing their susceptibility to substance use, while those with higher HL are better equipped to assess risks [32, 33]. HL supports critical evaluation of health information and informed decision-making, helping mitigate substance use risks [34]. Furthermore, evidence suggests that smoking and alcohol use correlate with poorer cognitive functioning and decision-making abilities, potentially impairing individuals’ capacity to understand, assess, and utilize health information effectively [35–37], highlighting HL’s role as a protective factor.

In terms of physical activity, our study found a significant association between physical activity and higher total HL scores, particularly in the health promotion sub-domain. This finding aligns with previous studies [38–40]. Engaging in physical activity fosters a deeper understanding and management of health information; notably, adolescents who exercise frequently are more likely to adopt health-promoting behaviors. This engagement enhances their motivation and capacity to seek, comprehend, and effectively apply health-related information, facilitating informed health choices and thus bolstering HL levels [39]. Furthermore, regular physical activity is linked to enhanced cognitive functioning and brain structure, which may support adolescents’ abilities to process and retain health information [41, 42]. Herting and Chu [41] suggest that exercise contributes to improved academic performance and cognitive functions, including attention, memory, and executive functions like problem-solving and inhibitory control. Exercise also appears to positively impact brain structure, especially in regions associated with learning and memory. Adolescence is a critical period for brain development and may be particularly sensitive to physical activity, suggesting that exercise during this time can have lasting benefits for brain health and function. HL equips individuals with the essential knowledge and skills needed for informed health decisions, including understanding the preventive benefits of physical activity for chronic disease and overall health improvement. Higher HL supports the effective interpretation of health information, encouraging proactive health behaviors like regular physical activity as part of a healthy lifestyle [40].

Regarding with dietary habits, our study found a significant association between the consumption of sweet foods/drinks and lower total HL scores, particularly in the healthcare and health promotion sub-domains. Conversely, no association was found between HL and the consumption of salty and fatty foods. This finding aligns with the systematic review by Buja et al. [43], which suggests that higher HL correlates with lower sugar intake, especially among adults. Specifically, adults with higher HL tend to consume fewer sugar-sweetened beverages. However, there is limited evidence to suggest a clear link between HL and reduced intake of salt and fat. This association may reflect limited awareness of high-sugar food risks, as sugar-related health issues are frequently highlighted in public health messaging [43, 44]. Social influences, social norms, and understanding of nutritional labelling likely contribute to this association. Regarding social norm, a study by Belanger-Gravel et al. [45] revealed that people who perceive high sugar-sweetened beverage consumption among their close contacts or society at large are more likely to consume sugar-sweetened beverage themselves. Understanding nutritional labelling further supports healthier choices [44, 46, 47]. Thai government initiatives, such as the “less-sweet-less-disease” campaign, GDA labels, and sugar taxes, have reduced sugary drink consumption, but expanding policies to other sugar sources is recommended. Persistent misconceptions, particularly about natural sugars, highlight the need for continuous, accurate public health communication [48].

### Implications for policy and practice

The findings from this study underscore the importance of targeted HL interventions within Thai university settings, particularly for non-health science students who may lack adequate exposure to health information in their academic curriculum. Implementing HL modules that address essential skills such as accessing, understanding, appraising, and applying health information could bridge the HL gap between health and non-health students. This strategy aligns with the evidence showing that health sciences students tend to achieve higher literacy levels, likely due to the increased health-related content in their courses. Universities could consider incorporating HL components, such as workshops, seminars, or integrated learning modules, into general education requirements to enhance students’ capacity to make informed health decisions. Moreover, the Thai government could expand its existing “Health Media for Children and Adolescents” initiative to include young adults and university students, addressing a significant gap in current HL promotion efforts. Extending this initiative would ensure that HL resources and interventions reach a broader demographic, fostering a more health-literate society. On a global scale, universities can draw from these findings to develop context-specific HL programs that consider the diverse sociodemographic and behavioral profiles of students. Integrating HL into higher education policies aligns with broader global health promotion strategies, such as those advocated by the World Health Organization, emphasizing the role of education in achieving health equity.

Additionally, the role of behavioral interventions cannot be overlooked. With significant associations observed between HL and behaviors such as smoking, alcohol use, physical activity, and diet, health promotion campaigns on campuses should integrate strategies that address these behaviors directly. Promoting HL awareness and strengthening media literacy skills would further equip students to navigate and critically assess health information. For instance, promoting physical activity as part of a holistic health education approach could reinforce both HL and overall wellness among students. Interventions aimed at improving nutritional literacy could empower individuals to make healthier dietary choices. By increasing awareness of the adverse health effects of high sugar consumption, such interventions could contribute to behavior change, encouraging a shift toward more balanced diets. Moreover, given the link between socioeconomic factors on HL, highlights the need for tailored interventions. Targeted support for students from lower socioeconomic backgrounds, such as financial assistance for health resources, and programs specifically addressing the HL needs of male students could effectively reduce disparities. The university clinic, located on campus, provides an excellent avenue for intervention development. Educational workshops and awareness campaigns could be hosted in collaboration with the clinic to address primary health concerns and foster health literacy. A multifaceted approach, incorporating digital, university, and community-based interventions should be implemented to enhance health literacy, with early, targeted efforts to empower young adults with HL skills necessary for health decision-making [49].

### Limitations and future research

While this study offers valuable insights for policy and practice in university health promotion, limitations should be acknowledged. This study’s cross-sectional design limits the ability to establish causality between HL and influencing factors among university students. Although associations were identified, longitudinal studies would be necessary to determine if these factors directly impact HL over time. Additionally, self-reported behaviors, such as smoking, alcohol consumption, and dietary habits, may be subject to social desirability bias, potentially leading to underreporting. Future research could benefit from objective measures or longitudinal designs to confirm these associations and minimize bias. The use of a multi-stage stratified random sampling method minimized potential sampling bias; however, the final sample may not fully represent the broader population of Thai university students. Students from smaller or less diverse institutions may have different health literacy levels and determinants not captured in this study. While the study adjusted for several known sociodemographic and behavioral factors, the influence of unmeasured confounders, such as mental health status, digital health literacy, and access to healthcare services, cannot be ruled out. These unmeasured variables may have influenced the observed relationships and should be considered in future studies. Moreover, this study was conducted at a single university, which, although the largest in northern Thailand, limits the generalizability of the findings to other institutions or regions with differing sociodemographic or cultural characteristics. Expanding the research to multiple universities or including diverse student groups could provide a broader understanding of HL determinants across different academic and cultural contexts. Future studies might also explore how tailored interventions that consider these sociodemographic and behavioral influences could enhance HL in specific student subgroups, ultimately promoting healthier lifestyles and reducing health disparities.

## Conclusion

Overall, this study highlights the substantial influence of academic discipline, gender, and lifestyle behaviors on HL among university students. The higher HL observed in health sciences students underscores the value of incorporating health education into non-health curricula. Targeted interventions to address smoking, alcohol use and promote physical activity may further enhance HL. Targeted support for students from lower socioeconomic backgrounds, and programs specifically addressing the HL needs of male students could effectively reduce disparities. These insights provide a foundation for future HL programs aimed at fostering a more health-literate student population, ultimately contributing to better individual and public health outcomes.

The government and relevant organizations have a vital role in enhancing HL among university students. They can achieve this by creating targeted policies, allocating financial resources for HL programs in higher education, and launching campaigns that promote preventive health measures and informed decision-making among young adults.

Universities also play a crucial role by integrating HL modules into their curricula, equipping students with the skills to access, understand, and use health information effectively. Additionally, providing accessible health services, counseling, and wellness programs on campus addresses students’ physical and mental health needs. Collaboration with primary and secondary schools will also be vital, as initiating health promotion interventions at an earlier stage can significantly enhance HL before students enter higher education. For example, integrating HL concepts into primary and secondary school curricula ensures a strong foundation for lifelong health literacy. Universities should also engage in ongoing research to assess student HL levels and evaluate the effectiveness of programs, fostering continuous improvement. Tailored HL interventions should focus on bridging the gap between health and non-health science students by addressing the unique challenges faced by the latter group. Incorporating digital tools, such as mobile apps and online platforms, could further enhance engagement and accessibility. Through collaboration, governments, schools, and universities can build supportive environments that enhance HL, leading to better health outcomes and more informed decisions among university students.

## Supplementary Information

Additional file 1. Questionnaire.

Additional file 2. Univariable analysis for investigating factors associated with health literacy in university students.

Additional file 3. Health literacy scores in individual HL items.

## Data Availability

The original contributions presented in the study are included in the article. Further inquiries can be directed to the corresponding author.

## Abbreviations

95% CI: 95% confidence interval
SD.: Standard deviation
β: Beta
SE.: Standard error
*: *p* value < 0.05
**: *p* value < 0.01
HL: Health literacy
