# Supplementary material for: Health literacy disparities in Thai university students: exploring differences between health science and non-health science disciplines
Source: BMC Public Health. 2025 Feb 11;25:557. doi: 10.1186/s12889-025-21761-0 (PMC11817181; doi:10.1186/s12889-025-21761-0)
Supplement: Supplementary file 1 — Additional file 1. Questionnaire. [file 12889_2025_21761_MOESM1_ESM.docx]

**Volunteer Code: _______________**

**Interview Form for Regular Undergraduate Students, Chiang Mai University**

**Research Project Title**: Factors affecting health literacy in undergraduate students at Chiang Mai University.

**Eligibility Criteria**: Currently enrolled as a regular undergraduate student a University.

**Section 1: Personal Information**

1. **Gender**
   ❒ 1) Male
   ❒ 2) Female
2. **Age**: _______ years
3. **Religion**
   ❒ 1) Buddhism
   ❒ 2) Christianity
   ❒ 3) Islam
   ❒ 4) Other
4. **Marital Status**
   ❒ 1) Single
   ❒ 2) In a relationship
   ❒ 3) Married
   ❒ 4) Divorced/Widowed
5. **Monthly Income**
   ❒ 1) Less than 5,000 THB/month
   ❒ 2) 5,000–10,000 THB/month
   ❒ 3) 10,000–15,000 THB/month
   ❒ 4) More than 15,000 THB/month
6. **Chronic Disease**
   ❒ 1) Yes
   ❒ 2) No
   If yes, specify: _______________
7. **Type of Residence**
   ❒ 1) Living with family
   ❒ 2) Living alone in a dormitory
   ❒ 3) Living in a dormitory with a roommate
8. **Current Area of Residence**
   ❒ 1) Outside Mueang District
   ❒ 2) Inside Mueang District
9. **Area of Residence Before Attending University**
   ❒ 1) Outside Mueang District
   ❒ 2) Inside Mueang District
10. **Physical activity**

❒ 1) Never

❒ 2) 1-2 days/week

❒ 3) 3 days/week

❒ 4) 4-5 days/week

❒ 5) 6-7 days/week

**Section 2: Educational Information**

1. **Faculty of Study**
   ❒ 01 Humanities
   ❒ 03 Fine Arts
   ❒ 05 Science
   ❒ 06 Engineering
   ❒ 07 Medicine
   ❒ 11 Medical Technology
   ❒ 12 Nursing
   ❒ 17 Architecture
   ❒ 19 Political Science and Public Administration
   ❒ 20 Law
2. **Year of Study**
   ❒ 1) Year 1
   ❒ 2) Year 2
   ❒ 3) Year 3
   ❒ 4) Year 4
3. **Parents’ Educational Level**
   ❒ 1) No formal education
   ❒ 2) Primary education
   ❒ 3) Secondary education
   ❒ 4) Bachelor's degree
   ❒ 5) Higher than a bachelor's degree
4. **Overall GPA**
   ❒ 1) GPAX < 1.5
   ❒ 2) GPAX 1.51–2.00
   ❒ 3) GPAX 2.01–2.50
   ❒ 4) GPAX 2.51–3.00
   ❒ 5) GPAX 3.01–3.50
   ❒ 6) GPAX 3.51–4.00

**Section 3: Health Service Utilization**

1. **Health Insurance Coverage**
   ❒ 1) Government/State Enterprise
   ❒ 2) Universal Health Coverage (Gold Card)
   ❒ 3) Private Health Insurance
   ❒ 4) Self-funded
2. **Illness in the Past 6 Months**
   ❒ 1) Yes
   ❒ 2) No
3. **Health Services Used When Seeking Treatment** (Choose more than one if applicable)
   ❒ 1) Let it heal on its own
   ❒ 2) Self-medicate
   ❒ 3) Consult a doctor
4. **Preferred Health Services When Ill** (Choose more than one if applicable)
   ❒ 1) Chiang Mai University Health Center
   ❒ 2) Public hospital
   ❒ 3) Private hospital
   ❒ 4) Clinic
   ❒ 5) Other: _______________
5. **Reasons for Choosing Health Services** (Choose more than one if applicable)
   ❒ 1) Eligibility for treatment at the facility
   ❒ 2) Good service quality
   ❒ 3) Convenience and speed
   ❒ 4) Easy to access

**Section 4: dietary habits**

1. Eating fatty foods

❒ 1) Never

❒ 2) 1-3 days/week

❒ 3) 4-7 days/week

1. Eating sweet drinks/foods

❒ 1) Never

❒ 2) 1-3 days/week

❒ 3) 4-7 days/week

1. Eating salty foods

❒ 1) Never

❒ 2) 1-3 days/week

❒ 3) 4-7 days/week

**Section 5: Health Literacy**

This section involves questions about your ability to find, understand, and use health-related information. Respond with the perceived difficulty:

| items | Questions | 1  Very difficult | 2  Difficult | 3  Easy | 4  Very easy |
| --- | --- | --- | --- | --- | --- |
| 1 | find information about symptoms of illnesses that concern you? |  |  |  |  |
| 2 | find information on treatments of illnesses that concern you? |  |  |  |  |
| 3 | find out what to do in case of a medical emergency? |  |  |  |  |
| 4 | find out where to get professional help when you are ill? |  |  |  |  |
| 5 | understand what your doctor says to you? |  |  |  |  |
| 6 | understand the leaflets that come with your medicine? |  |  |  |  |
| 7 | understand what to do in a medical emergency? |  |  |  |  |
| 8 | understand your doctor’s or pharmacist’s instruction on how to take a prescribed medicine? |  |  |  |  |
| 9 | judge how information from your doctor applies to you? |  |  |  |  |
| 10 | judge the advantages and disadvantages of different treatment options? |  |  |  |  |
| 11 | judge when you may need to get a second opinion from another doctor? |  |  |  |  |
| 12 | judge if the information about illness in the media is reliable? |  |  |  |  |
| 13 | use information the doctor gives you to make decisions about you illness? |  |  |  |  |
| 14 | follow the instructions on medication? |  |  |  |  |
| 15 | call an ambulance in an emergency? |  |  |  |  |
| 16 | follow instructions from your doctor or pharmacist? |  |  |  |  |
| 17 | find information about how to manage unhealthy behaviour such as smoking, low physical activity and drinking too much? |  |  |  |  |
| 18 | find information on how to manage mental health problems like stress or depression? |  |  |  |  |
| 19 | find information about vaccinations and health screenings that you should have? |  |  |  |  |
| 20 | find information on how to prevent or manage conditions like being overweight, high blood pressure or high cholesterol |  |  |  |  |
| 21 | understand health warnings about behaviour such as smoking, low physical activity and drinking too much? |  |  |  |  |
| 22 | understand why you need vaccinations? |  |  |  |  |
| 23 | understand why you need health screenings? |  |  |  |  |
| 24 | judge how reliable health warnings are, such as smoking, low physical activity and drinking too much? |  |  |  |  |
| 25 | judge when you need to go to a doctor for a check-up? |  |  |  |  |
| 26 | judge which vaccinations you may need? |  |  |  |  |
| 27 | judge which health screenings you may have? |  |  |  |  |
| 28 | judge if the information on health risks in the media is reliable? |  |  |  |  |
| 29 | decide if you should have a flu vaccination? |  |  |  |  |
| 30 | decide how you can protect yourself from illness based on advice from family and friends? |  |  |  |  |
| 31 | decide how you can protect yourself from illness based on information in the media? |  |  |  |  |
| 32 | find information on healthy activities such as exercise healthy food and nutrition? |  |  |  |  |
| 33 | find out about activities that are good for your mental well-being? |  |  |  |  |
| 34 | find information on how your neighbourhood could be more health-friendly? |  |  |  |  |
| 35 | find out about political changes that may affect health? |  |  |  |  |
| 36 | find out about efforts to promote your health at work, at school or in the community? |  |  |  |  |
| 37 | understand advice on health from family members or friends? |  |  |  |  |
| 38 | understand information on food packaging? |  |  |  |  |
| 39 | understand information in the media on how to get healthier? |  |  |  |  |
| 40 | understand information on how to keep your mind healthy? |  |  |  |  |
| 41 | judge where your life affects your health and wellbeing? |  |  |  |  |
| 42 | judge how your housing conditions help you to stay healthy? |  |  |  |  |
| 43 | judge which everyday behaviour is related to your health? |  |  |  |  |
| 44 | make decisions to improve your health? |  |  |  |  |
| 45 | join a sports club or exercise class if you want to? |  |  |  |  |
| 46 | influence your living conditions that affect your health and wellbeing? |  |  |  |  |
| 47 | take part in activities that improve health and wellbeing? |  |  |  |  |
