# Supplementary material for: Health literacy disparities in Thai university students: exploring differences between health science and non-health science disciplines
Source: BMC Public Health. 2025 Feb 11;25:557. doi: 10.1186/s12889-025-21761-0 (PMC11817181; doi:10.1186/s12889-025-21761-0)
Supplement: Supplementary file 2 — Additional file 2. Univariable analysis for investigating factors associated with health literacy in university students. [file 12889_2025_21761_MOESM2_ESM.docx]

**Supplementary 2.** Univariable analysis for investigating factors associated with health literacy in university students

| **Parameters** |  | **n** | **Total HL** | **Sub-domain1:**  **Healthcare** | **Sub-domain2:**  **Disease prevention** | **Sub-domain3: Health promotion** |
| --- | --- | --- | --- | --- | --- | --- |
| Gender | Male | 555 | 37.5+7.7 ** | 37.0+8.3** | 37.7+8.9** | 37.8+8.7** |
|  | Female | 1,092 | 39.5+7.1 | 39.4+7.1 | 40.2+8.1 | 38.8+8.5 |
| Religion | Buddhist | 1,547 | 38.8+7.4 | 38.6+7.6 | 39.4+8.5 | 38.5+8.5 |
|  | Others | 100 | 38.4+7.6 | 38.1+8.0 | 39.2+8.9 | 37.8+9.6 |
| Relationship status | Single | 1,149 | 38.6+7.5 | 38.4+7.7 | 39.2+8.6 | 38.3+8.7 |
|  | Boyfriend/girlfriend | 498 | 39.2+7.1 | 39.0+7.5 | 39.8+8.2 | 38.8+8.3 |
| Monthly income | <150 USD/month | 592 | 37.8+7.5**^ab,ac,bc^ | 37.7+7.3** ^ab,ac,bc^ | 38.2+8.5** ^ab,ac,bc^ | 37.6+9.0**^ab,ac^ |
|  | 150-300 USD/month | 819 | 39.1+7.3 | 38.7+7.8 | 39.7+8.4 | 38.8+8.4 |
|  | >300 USD/month | 236 | 40.3+7.0 | 40.3+7.5 | 41.0+8.2 | 39.8+8.2 |
| Living status | With family | 189 | 38.6+7.5 | 38.7+7.3 | 39.1+8.7 | 38.1+9.0 |
|  | alone | 703 | 38.8+7.4 | 38.5+7.7 | 39.7+8.6 | 38.4+8.7 |
|  | With roommate | 755 | 38.8+7.3 | 38.7+7.6 | 39.2+8.4 | 38.6+8.4 |
| Parental education | Primary school or lesser | 246 | 37.9+7.9*ac | 37.9+8.1 | 38.3+9.0**^ac,bc^ | 37.6+9.0 |
|  | Secondary school | 499 | 38.5+7.3 | 38.5+7.3 | 38.8+8.3 | 28.2+8.4 |
|  | Bachelor degree or higher | 902 | 39.2+7.3 | 38.8+7.7 | 40.0+8.4 | 28.9+8.6 |
| Hometown status | Rural community | 1,012 | 38.7+7.3 | 38.5+7.7 | 39.2+8.5 | 38.5+8.5 |
|  | Urban Community | 635 | 38.9+7.5 | 38.7+7.4 | 39.7+8.5 | 38.5+8.8 |
| Comorbidity | Yes | 159 | 38.8+7.3 | 39.3+6.9 | 39.4+8.7 | 37.6+8.7 |
|  | No | 1488 | 38.8+7.4 | 38.5+7.7 | 39.4+8.5 | 38.6+8.6 |
| History illness in past 6 months | Yes | 920 | 38.8+7.4 | 38.7+7.6 | 39.3+8.6 | 38.4+8.7 |
|  | No | 727 | 38.9+7.4 | 38.5+7.6 | 39.5+8.4 | 38.6+8.5 |
| Smoking | Never ^a^ | 1,486 | 39.2+7.2**^ab,ac,ad,ae,de^ | 36.2+7.10**^ab,ac,ad,ae,ce,de^ | 36.2+7.15**^ab,ac,ad,ae^ | 36.2+7.20**^ac,ad^ |
|  | 1-2days/week ^b^ | 42 | 36.2+7.6 | 36.2+7.11 | 36.2+7.16 | 36.2+7.21 |
|  | 3days/week ^c^ | 40 | 36.2+7.7 | 36.2+7.12 | 36.2+7.17 | 36.2+7.22 |
|  | 4-5days/week ^d^ | 24 | 36.2+7.8 | 36.2+7.13 | 36.2+7.18 | 36.2+7.23 |
|  | 6-7days/week ^e^ | 55 | 36.2+7.9 | 36.2+7.14 | 36.2+7.19 | 36.2+7.24 |
| Alcohol consumption | Never ^a^ | 793 | 39.5+7.3**^ac,ad,ae,bc,bd,be^ | 39.2+7.4**^ac,ad,ae,bc,bd,be^ | 40.3+8.2**^ab,ac,ad,bc,bd^ | 39.1+8.6**^ac,ad,ae,bc,bd^ |
|  | 1-2days/week ^b^ | 611 | 39.0+7.0 | 38.9+7.2 | 39.3+8.4 | 38.7+8.3 |
|  | 3days/week ^c^ | 158 | 36.1+7.9 | 36.1+7.9 | 36.1+8.5 | 36.0+9.2 |
|  | 4-5days/week ^d^ | 51 | 36.0+8.1 | 36.1+8.7 | 36.1+9.6 | 35.8+8.6 |
|  | 6-7days/week ^e^ | 34 | 36.0+8.4 | 34.4+11.1 | 37.6+9.3 | 36.0+9.3 |
| Physical activity | Never ^a^ | 395 | 38.0+7.5**^ab,ac,cd,ce^ | 38.4+7.3**^ac,bd,be,cd,ce^ | 39.1+8.5 | 36.6+9.2**^ab,ac,ad,ae^ |
|  | 1-2days/week ^b^ | 590 | 39.1+7.0 | 38.8+7.2 | 39.8+8.1 | 38.8+8.2 |
|  | 3days/week ^c^ | 339 | 39.8+6.9 | 39.6+7.2 | 39.9+8.0 | 39.8+7.8 |
|  | 4-5days/week ^d^ | 205 | 38.2+8.4 | 37.4+9.0 | 38.5+9.6 | 38.7+9.1 |
|  | 6-7days/week ^e^ | 118 | 38.2+8.1 | 37.3+8.6 | 38.0+9.4 | 39.2+9.3 |
| Dietary habits-Fatty foods | Never ^a^ | 26 | 40.08+6.64*^bc^ | 41.51+6.77*^ac^ | 38.10+9.09*^bc^ | 40.68+7.48**^bc^ |
|  | 1-3 days/week ^b^ | 960 | 39.21+7.22 | 38.82+7.49 | 38.95+8.40 | 39.92+8.27 |
|  | 4-7days/week ^c^ | 661 | 38.16+7.63 | 38.13+7.78 | 37.85+8.86 | 38.51+8.78 |
| Dietary habits-Sweet foods/drinks | Never ^a^ | 99 | 40.16+6.93*^ac,bc^ | 40.32+6.45*^ab,ac,bc^ | 39.54+9.02**^ac,bc^ | 40.66+8.71 |
|  | 1-3 days/week ^b^ | 924 | 39.06+7.15 | 38.67+7.51 | 39.00+8.31 | 39.53+8.19 |
|  | 4-7days/week ^c^ | 624 | 38.21+7.77 | 38.19+7.88 | 37.58+8.91 | 38.91+8.86 |
| Dietary habits-Salty foods | Never ^a^ | 298 | 39.85+7.12*^ab,ac^ | 39.65+7.19*^ab,ac^ | 39.42+8.58*^ac^ | 40.51+8.33*^ab,ac^ |
|  | 1-3 days/week ^b^ | 991 | 38.63+7.15 | 38.40+7.36 | 38.41+8.44 | 39.13+8.25 |
|  | 4-7days/week ^c^ | 358 | 38.40+8.17 | 38.22+8.51 | 37.96+9.05 | 39.07+9.18 |
| Faculty studying | Non-health sciences | 971 | 37.3+7.6** | 37.2+7.8** | 37.6+8.7** | 37.2+8.8** |
|  | Health sciences | 676 | 40.9+6.6 | 40.6+6.8 | 41.9+7.5 | 40.4+8.0 |
| Year of study | 1^st^ year | 428 | 38.8+7.8 | 38.5+7.9 | 39.2+8.8 | 38.9+8.9 |
|  | 2^nd^ year | 426 | 38.3+7.2 | 39.4+7.5 | 39.1+8.4 | 38.0+8.7 |
|  | 3^rd^ year | 372 | 39.3+6.9 | 39.4+7.2 | 39.6+8.2 | 38.9+8.0 |
|  | 4^th^ year | 421 | 38.8+7.5 | 38.7+7.8 | 39.6+8.5 | 38.2+8.7 |
| GPAX | <2.50 ^a^ | 329 | 37.4+7.2**^ab,ac,bc^ | 37.4+7.8**^ab,ac^ | 37.5+8.4**^ab,ac,bc^ | 37.4+8.6**^ac,bc^ |
|  | 2.51-3.50 ^b^ | 736 | 38.7+7.6 | 38.6+7.6 | 39.3+8.6 | 38.3+8.7 |
|  | >3.51 ^c^ | 582 | 39.7+7.2 | 39.3+7.4 | 40.5+8.3 | 39.4+8.4 |

* *p* value <0.05; ** *p* value < 0.01
