# Supplementary material for: Health literacy disparities in Thai university students: exploring differences between health science and non-health science disciplines
Source: BMC Public Health. 2025 Feb 11;25:557. doi: 10.1186/s12889-025-21761-0 (PMC11817181; doi:10.1186/s12889-025-21761-0)
Supplement: Supplementary file 3 — Additional file 3. Health literacy scores in individual HL items. [file 12889_2025_21761_MOESM3_ESM.docx]

**Supplementary 3.** Health literacy scores in individual HL items

| **Question no.** | **Total**  **(n=1647)** | **Health science students (n=676)** | **Non-health science students (n=971)** | ***p* value** |
| --- | --- | --- | --- | --- |
| 1.find information about symptoms of illnesses that concern you? | 3.20+0.67 | 3.36+0.63 | 3.09+0.68 | ** |
| 2. find information on treatments of illnesses that concern you? | 3.20+0.67 | 3.32+0.64 | 3.12+0.67 | ** |
| 3. find out what to do in case of a medical emergency? | 3.16+0.70 | 3.25+0.66 | 3.09+0.73 | ** |
| 4. find out where to get professional help when you are ill? | 3.29+0.72 | 3.42+0.67 | 3.20+0.73 | ** |
| 5. understand what your doctor says to you? | 3.43+0.65 | 3.57+0.56 | 3.33+0.69 | ** |
| 6. understand the leaflets that come with your medicine? | 3.44+0.68 | 3.57+0.61 | 3.34+0.71 | ** |
| 7. understand what to do in a medical emergency? | 3.12+0.76 | 3.23+0.72 | 3.04+0.78 | ** |
| 8. understand your doctor’s or pharmacist’s instruction on how to take a prescribed medicine? | 3.54+0.61 | 3.64+0.54 | 3.48+0.64 | ** |
| 9. judge how information from your doctor applies to you? | 3.47+0.63 | 3.58+0.57 | 3.39+0.66 | ** |
| 10. judge the advantages and disadvantages of different treatment options? | 3.21+0.75 | 3.32+0.73 | 3.13+0.76 | ** |
| 11. judge when you may need to get a second opinion from another doctor? | 3.06+0.82 | 3.19+0.79 | 2.98+0.83 | ** |
| **12. judge if the information about illness in the media is reliable?** | **3.03+0.82** | **3.19+0.78** | **2.92+0.83** | ****** |
| 13. use information the doctor gives you to make decisions about your illness? | 3.30+0.67 | 3.42+0.60 | 3.22+0.70 | ** |
| 14. follow the instructions on medication? | 3.64+0.59 | 3.74+0.50 | 3.56+0.64 | ** |
| 15. call an ambulance in an emergency? | 3.35+0.80 | 3.48+0.70 | 3.26+0.85 | ** |
| 16. follow instructions from your doctor or pharmacist? | 3.61+0.60 | 3.71+0.51 | 3.54+0.66 | ** |
| 17. find information about how to manage unhealthy behavior such as smoking, low physical activity and drinking too much? | 3.54+0.64 | 3.66+0.55 | 3.45+0.68 | ** |
| 18. - find information on how to manage mental health problems like stress or depression? | 3.31+0.76 | 3.45+0.67 | 3.21+0.80 | ** |
| 19. find information about vaccinations and health screenings that you should have? | 3.22+0.79 | 3.37+0.74 | 3.11+0.80 | ** |
| 20. find information on how to prevent or manage conditions like being overweight, high blood pressure or high cholesterol? | 3.35+0.70 | 3.51+0.62 | 3.24+0.72 | ** |
| 21. understand health warnings about behavior such as smoking, low physical activity and drinking too much? | 3.51+0.66 | 3.65+0.57 | 3.41+0.70 | ** |
| 22. understand why you need vaccinations? | 3.64+0.59 | 3.78+0.48 | 3.55+0.64 | ** |
| 23. understand why you need health screenings? | 3.64+0.60 | 3.79+0.47 | 3.54+0.66 | ** |
| 24. judge how reliable health warnings are, such as smoking, low physical activity and drinking too much? | 3.48+0.66 | 3.62+0.59 | 3.39+0.69 | ** |
| 25. judge when you need to go to a doctor for a check-up? | 3.35+0.77 | 3.51+0.67 | 3.24+0.81 | ** |
| 26. judge which vaccinations you may need? | 3.16+0.87 | 3.39+0.77 | 3.01+0.91 | ** |
| 27. judge which health screenings you should have? | 3.16+0.84 | 3.35+0.77 | 3.03+0.86 | ** |
| 28. judge if the information on health risks in the media is reliable? | 3.19+0.80 | 3.37+0.74 | 3.07+0.83 | ** |
| 29. decide if you should have a flu vaccination? | 3.36+0.78 | 3.53+0.68 | 3.24+0.82 | ** |
| 30. how you can protect yourself from illness based on advice from family and friends? | 3.29+0.74 | 3.41+0.69 | 3.21+0.77 | ** |
| 31. decide how you can protect yourself from illness based on information in the media? | 3.21+0.78 | 3.35+0.73 | 3.12+0.79 | ** |
| 32. find information on healthy activities such as exercise, healthy food and nutrition? | 3.56+0.60 | 3.67+0.52 | 3.48+0.63 | ** |
| 33. find out about activities that are good for your mental well-being? | 3.48+0.67 | 3.61+0.59 | 3.39+0.71 | ** |
| **34. find information on how your friends could be more health-friendly?** | **2.99+0.92** | **3.10+0.91** | **2.91+0.92** | ****** |
| 35. find out about political changes that may affect health? | 3.10+0.85 | 3.18+0.85 | 3.05+0.85 | ** |
| 36. find out about efforts to promote your health at university? | 3.17+0.79 | 3.30+0.77 | 3.09+0.79 | ** |
| 37. understand advice on health from family members or friends? | 3.41+0.67 | 3.56+0.58 | 3.31+0.71 | ** |
| 38. understand information on food packaging? | 3.37+0.72 | 3.52+0.63 | 3.26+0.75 | ** |
| 39. understand information in the media on how to get healthier? | 3.47+0.63 | 3.62+0.56 | 3.37+0.66 | ** |
| 40. understand information on how to keep your mind healthy? | 3.46+0.66 | 3.59+0.60 | 3.36+0.69 | ** |
| 41. judge where your life affects your health and well-being? | 3.40+0.72 | 3.53+0.67 | 3.32+0.74 | ** |
| 42. judge how your housing conditions help you to stay healthy? | 3.45+0.68 | 3.57+0.63 | 3.37+0.71 | ** |
| 43. judge which everyday behavior is related to your health? | 3.51+0.65 | 3.63+0.58 | 3.42+0.68 | ** |
| 44. make decisions to improve your health? | 3.32+0.75 | 3.40+0.74 | 3.26+0.76 | ** |
| 45. join a sports club or exercise class if you want to? | 3.06+0.93 | 3.11+0.92 | 3.03+0.94 |  |
| 46. influence your living conditions that affect your health and wellbeing? | 3.25+0.78 | 3.35+0.76 | 3.18+0.78 | ** |
| **47. take part in activities that improve health and well-being in your university?** | **2.95+0.97** | **3.04+0.97** | **2.89+0.97** | ****** |

** *p* value < 0.01
